# Acute Stressor Exposure Modifies Plasma Exosome-Associated Heat Shock Protein 72 (Hsp72) and microRNA (miR-142-5p and miR-203)

**DOI:** 10.1371/journal.pone.0108748

**Published:** 2014-09-26

**Authors:** Lida A. Beninson, Peter N. Brown, Alice B. Loughridge, Jonel P. Saludes, Thomas Maslanik, Abigail K. Hills, Tyler Woodworth, Wendy Craig, Hang Yin, Monika Fleshner

**Affiliations:** 1 Department of Integrative Physiology and the Center for Neuroscience, University of Colorado at Boulder, Boulder, Colorado, United States of America; 2 Department of Chemistry and Biochemistry and the BioFrontiers Institute, University of Colorado at Boulder, Boulder, Colorado, United States of America; 3 Department of Chemistry, Washington State University, Pullman, Washington, United States of America; 4 Center of Basic Molecular Science, Department of Chemistry, Tsinghua University, Beijing, China; University of Arkansas for Medical Sciences, United States of America

## Abstract

Exosomes, biologically active nanoparticles (40–100 nm) released by hematopoietic and non-hematopoietic cells, contain a variety of proteins and small, non-coding RNA known as microRNA (miRNA). Exposure to various pathogens and disease states modifies the composition and function of exosomes, but there are no studies examining *in* *vivo* exosomal changes evoked by the acute stress response. The present study reveals that exposing male Fisher 344 rats to an acute stressor modulates the protein and miRNA profile of circulating plasma exosomes, specifically increasing surface heat shock protein 72 (Hsp72) and decreasing miR-142-5p and -203. The selected miRNAs and Hsp72 are associated with immunomodulatory functions and are likely a critical component of stress-evoked modulation of immunity. Further, we demonstrate that some of these stress-induced modifications in plasma exosomes are mediated by sympathetic nervous system (SNS) activation of alpha-1 adrenergic receptors (ADRs), since drug-mediated blockade of the receptors significantly attenuates the stress-induced modifications of exosomal Hsp72 and miR-142-5p. Together, these findings demonstrate that activation of the acute stress response modifies the proteomic and miRNA profile of exosomes released into the circulation.

## Introduction

The extracellular environment within multicellular organisms contains unique nanoparticles (40–100 nm) known as exosomes. Exosomes are secreted into the extracellular environment and contain molecules unique to their cellular origin [1], [2]. Interest in the immunological roles of exosomes has grown rapidly since the discovery that exosomes modulate immunity through expression of MHC class I and II molecules [3], activation of natural killer cells [4], stimulation of T cells [5], and induction of tolerance to oral antigens [6]. Exposure to a variety of pathogens or diseases, such as microbial infection [7] and cancer [8], modifies the composition and function of exosomes, providing insight into their potential uses as biomarkers and therapeutic tools. While a growing body of evidence demonstrates that exosomes can be modified as a consequence of pathological or disease states, to date there are few studies examining proteomic and miRNA changes in circulating plasma exosomes after physiological challenges such as the systemic stress response.

Exposure to stressors, whether acute or chronic, can exert adverse consequences on immunity. Research has demonstrated that stressor exposure can exacerbate cardiovascular disease [9], diabetes [10], and cancer [11]. Additionally, research in stress physiology revealed its immunosuppressive impact through restrained T cell dependent antibody responses [12] and suppression of anti-viral host-defense [13]. In contrast, recent research demonstrates that exposure to acute stressors can potentiate innate immunity. Acute stressors can evoke exaggerated inflammatory cytokine and chemokine responses under sterile conditions [14], enhance dermatological immunity [15], and improve host defense to subcutaneous *Escherichia coli* (*E. coli*) challenge [16]. Potentiation of innate immunity could be an adaptive feature of the acute stress response, possibly enhancing an organism’s response to injuries sustained from the stressor. The mechanisms responsible for stress-modified immunity are still under investigation, but recent research on the immunological function of exosomes during disease make them a novel target for immunomodulation during the acute stress response.

To understand how acute stressor exposure might modify the immunological function of circulating exosomes, we examined components of the stress response that could be likely candidates for both associating with exosomes and modulating immunity. Of particular interest is the 72 kD heat shock protein (Hsp72), a molecular chaperone abundant in the plasma following acute stressor exposure [16]. When exposed to a stressor, cells synthesize intracellular Hsp72 to maintain cellular homeostasis by refolding denatured proteins and promoting cellular survival [17]. However, when cells release Hsp72 into the extracellular environment, it becomes immunologically active by stimulating leukocytes through toll like receptors (TLR) and inducing the secretion of pro-inflammatory molecules [18]. The mechanism of Hsp72 release into the circulation during stressor exposure is unclear, but several studies have demonstrated that Hsp72 can associate with exosomes in cell culture [19]–[22] and amniotic fluid [23]. Thus far, it is unknown whether stress-induced Hsp72 circulating in the plasma is associated with exosomes. Given the reported immunological properties of both exosomes and extracellular Hsp72, it is important to determine if stressor exposure modifies host immunity by increasing Hsp72 associated with plasma exosomes.

Exosomes also contain non-coding RNA known as microRNA (miRNA) [24], [25]. Exosomal miRNA can elicit activity on distal cells upon inclusion [25], regulating target genes and modulating translation of messenger RNA (mRNA). Various environmental stressors, such as heat [26] and oxidative stress [27], can modify miRNA associated with TLR mediated inflammation [28], pro-inflammatory cytokine expression [29], and macrophage differentiation [30], making miRNA another target of interest in stress-modified immunity. To explore the impact of acute stress on plasma exosome miRNA, we analyzed miR-142-5p, -150, -155, and -203 based on evidence of their differential presence in heat stressed rats [26], involvement in TLR-mediated immunity [28], and association with macrophage differentiation [30].

We hypothesized that acute stressor exposure modulates the protein and miRNA character of circulating plasma exosomes. Our findings demonstrate that stress modifies plasma exosomes through up-regulation of surface Hsp72 and down-regulation of two miRNAs. Pathway enrichment analysis of the stress-modified exosomal miRNAs target genes reveals functionally enriched pathways implicated in the stress response. Further, we identify sympathetic nervous system (SNS) activation of the α_1_-adrenergic receptor (ADR) as an important signaling process to exosomal elevations of Hsp72 and down-regulation of miR-142-5p. These are the first studies to demonstrate that activation of the stress response modifies plasma exosomes that may be capable of regulating immunity.

## Materials and Methods

### Animals

Adult male Fisher 344 rats (8–9 weeks old; Harlan) weighing 230–275 grams were singly housed in Plexiglass Nalgene plastic cages (48×27×20 cm) with microisolator tops in a pathogen-free barrier facility and allowed access to food and water *ad libitum* (Harlan Standard Lab Chow). The Fisher rat is a highly stress responsive inbred rat strain chosen for this study due to their robust and consistent stress response, allowing us to use fewer animals per group. All experimental groups started with n = 8 per experimental condition, but animals were considered outliers and dropped from the study if their results failed the Grubbs Test of Outliers. Following arrival at the University of Colorado’s Transgenic Animal Facility (room temperature maintained at 23°C), rats were given two weeks to acclimate to the colony room prior to experimental procedures. Rats were handled briefly each day for one week prior to experimental procedures. The use of animals is necessary in this study because of the nature of information sought and the need for controlled conditions. This study was carried out in strict accordance with the recommendations in the National Research Council’s Guide for the Care and Use of Laboratory Animals. Care and use of the rats were in accordance with protocols (IACUC 1304.03: Extracellular heat shock protein 72 is a Danger Associated Molecular Pattern (DAMP) released by stress) reviewed and approved by the University of Colorado Institutional Care and Use Committee. All efforts were made to minimize suffering.

### Inescapable tail shock and plasma collection

Inescapable tail shock (stress) was performed as previously described [14]. Briefly, electrodes were placed across the tails protruding from the back of a restraining tube. Rats were exposed to 100, 1.5 mA, 5-second, intermittent, (average inter-trial interval of 60+/−25 s) tail shocks administered by an automated shock system (Colbourn Instruments). This stress procedure is a well-established model of acute stressor exposure that has been well characterized in both stress and immune responses. Previous research has demonstrated that Fisher 344 rats exposed to 15, 25, and 100 tail shocks have significantly elevated plasma Hsp72 in a time-dependent manner compared to non-stressed controls, and those concentrations rapidly decline one hour following stressor termination [31]. To analyze plasma exosome concentrations of Hsp72 at their peak elevation in the plasma, rats were sacrificed via rapid decapitation immediately following stressor termination. Trunk blood was collected in EDTA tubes (13×75 mm) and plasma was isolated at 3000×*g* at 4°C for 15 min. Spleens were aseptically removed and weighed. This stressor consistently elevates Hsp72 and inflammatory cytokines in the plasma [14].

### Exosome enrichment with ExoQuick

ExoQuick (Systems Biosciences) was used for plasma exosome isolation according to the manufacturer’s instructions. Plasma supernatant was aspirated and labeled “exosome depleted.” The precipitate was washed, re-suspended in 75 µl of PBS, and labeled “exosome enriched.” Exosome fractions used for visualization and Dynabead immunoprecipitation were derived from plasma pre-treated with Thromboplastin-D (ThermoScientific), an anti-coagulant, prior to ExoQuick precipitation to remove aggregating fibrins and fibrinogens. Briefly, 100 µL aliquots of the plasma were warmed to 37°C, treated with an equal volume of Thromboplastin-D, and incubated for 15 min. Fibrins and fibrinogens were pelleted from the plasma following centrifugation at 10,000 rpm for 5 min. The supernatant was fractionated with ExoQuick as described above.

### Exosome Visualization

Exosome enriched fractions from Thromboplastin-D treated plasma and untreated plasma were deposited on the surface of Formvar-carbon grids, air-dried, stained with uranyl acetate, and transmission election microscope (TEM) images were recorded with a Phillips CM100 transmission electron microscope (FEI, Inc.).

For nanoparticle tracking analysis (NTA), Thromboplastin-D treated plasma and exosome enriched samples were diluted 1∶10,000 while exosome depleted samples were diluted 1∶10 in sterile filtered PBS. NTA were performed using the LM10-HS instrument (NanoSight Ltd.) equipped with a green 532 nm laser in light scatter mode, with NTA 2.3 analytical software. The instrument was calibrated using commercially available 100 nm polystyrene beads (Polysciences) and sterile filtered PBS.

### ELISAs

Multiple markers (CD63, A33, IL-6) were measured in the exosome enriched and depleted fractions with commercially available ELISAs to determine successful exosome isolation. CD63 is a membrane tetraspanin common to exosomes from a variety of cellular sources [2], [32]. Intestinal epithelial cells release exosomes associated with A33, a protein not found in soluble form in the plasma [33]. Exosomes are not known to carry or transport IL-6, therefore, it should not be present in the exosome enriched fractions. Samples were run neat in the A33 (Uscn) and IL-6 ELISAs (R&D Systems). Exosome fractions were diluted 1∶20 for the CD63 ExoELISA (System Biosciences). Optical densities were measured using a SpectraMax Plus 354 plate reader (Molecular Devices) and analyzed using four-parameter curve fit and software (SoftMax 5.4.1). All values were corrected by total protein using a BCA ELISA (ThermoScientific) according to the manufacturer’s instructions. An additional assay using two common markers for exosomes, CD63 (BD Pharmingen; clone AD1, Cat. 551458, Lot 74411, 8 µg/mL) and the membrane transport protein Rab5b (Santa Cruz; rabbit polyclonal IgG, clone A-20, Cat. sc-598, lot H1810, 4 µg/mL), provides additional evidence for successful plasma exosome isolation [34]. Hsp72 was measured with a commercially available ELISA kit (Enzo Biosciences) according to the manufacturer’s instructions.

### Membrane Disruption of Exosomes

To confirm whether Hsp72 is associated with the surface membrane of plasma exosomes or contained within their lumen, the exosome membranes were ruptured prior to analysis. Following confirmation of the presence of exosomes with ExoELISA, the resuspended exosome enriched fraction was divided into a lysed group and a not lysed group. The exosome fraction in the lysed group was treated with an equal volume of exosome binding buffer (generously provided by System Biosciences) and subsequently warmed in a 37°C bath for 30 min. Exosome binding buffer is a proprietary detergent in 25 mM bicine buffer, pH 7.6. The exosome fraction from the not lysed group was left undisturbed but diluted in an equal volume of PBS. Both the lysed and not lysed exosome enriched fractions were immediately assayed for Hsp72 and BCA.

### CD63 immunoprecipitation

To examine CD63 depletion on Hsp72 concentrations, plasma was treated with Dynabeads (Invitrogen) coated in 50 µg/mL purified mouse anti-rat CD63 antibody (BD Pharmingen; clone AD1, Cat. 551458, Lot 74411) according to manufacturer’s instructions. Following CD63 immunoprecipitation, exosomes were precipitated with ExoQuick as described above. Fractions were analyzed for BCA, CD63 and Hsp72.

### α_1_-ADR antagonist administration

To determine whether blockade of the α_1_-ADR impacts exosome associated Hsp72 and miRNA following stressor exposure, the antagonist, prazosin (Sigma-Aldrich), was administrated to rats as previously described [35]. 30 min prior to tail shock, rats received 2.0 mg/kg i.p. injection of prazosin dissolved in endotoxin-free water or no injection. Pilot studies indicate that saline injection has no impact on cytokine, CD63 exosome, or Hsp72 concentrations compared to rats receiving no injection. Following stressor termination, rats were sacrificed and plasma and spleens were collected. Lactate dehydrogenase (LDH), a marker of cell death, was measured in the plasma using a commercial assay (Bioo Scientific) according to the manufacturer’s instruction. Plasma exosomes were isolated with ExoQuick and assayed for Hsp72, BCA, and CD63. Exosomal miRNA was subsequently analyzed as described below.

### miRNA quantification

Plasma exosome RNA was purified using SeraMir (System Biosciences). Isolated miRNA was reverse transcribed for qRT-PCR using the TaqMan miRNA Reverse Transcription Kit and miRNA-specific stem-loop primers (Applied BioSystems). MiRNA from stressed, control, stress + prazosin, and control + prazosin rats were analyzed for changes in miR-142-5p, miR-150, miR-155, and miR-203. Analysis was performed on the reverse transcript from 1.2 ng RNA. All real time qPCR analysis was performed in a 96-well plate (Bio-Rad) on the CFX96 (Bio-Rad) with TaqMan probes. Samples were run at a 95°C melting step for 15 sec and 60°C annealing and elongation step for 60 sec for 40 cycles. Samples were normalized to miR-191 as a stable endogenous control. Ct values were obtained using CFX manager 2.0 (Bio-Rad) and all fell within the limit of detection. Data were deconvoluted using the ΔΔCt method with samples normalized from RNA of exosomes isolated from control rats.

### Statistical analysis

Two-way ANOVAs were used to analyze the effect of stress on individual proteins. If significant interactions were present, Fisher’s protected least significant difference (PLSD) post-hoc analyses were conducted. Data are presented as means ± the standard error of the mean (SEM). *P*<0.05 was considered statistically significant.

## Results

### Visualization and characterization of plasma exosomes

**Figure 1:**
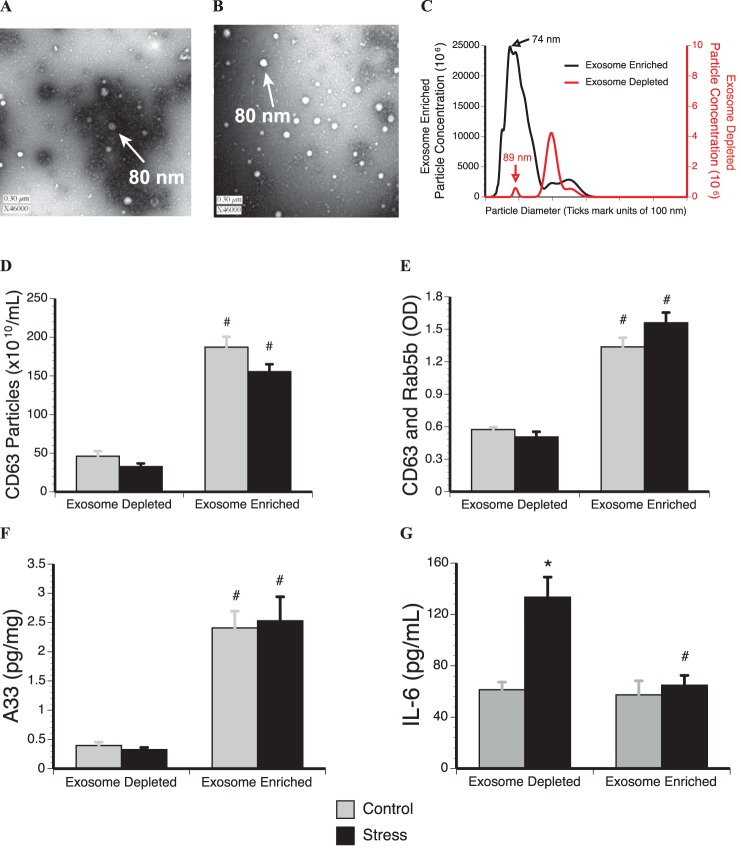
Confirmation of successful plasma exosome enrichment from male Fisher 344 rats. A. TEM demonstrates successful isolation of exosome-sized particles (∼80 nm) from the plasma with ExoQuick precipitation. B. TEM shows how pre-treating plasma with Thromboplastin-D prior to exosome isolation with ExoQuick removes clouding factors and prevents vesicle aggregation. C. Exosome enriched and exosome depleted plasma fractions from stressed rats pre-treated with Thromboplastin-D were characterized for size distribution and quantitated by NanoSight LM10, using light scatter from a 532 nm green laser. ELISA analyses reveal that ExoQuick exosome isolation successfully enriches exosomes as marked by known exosome markers. Exposing rats to tail shock stress (Stress) had no impact on the rate of plasma exosome release compared to non-stressed rats (Control) as measured by: D. the tetraspanin CD63, E. the adhesion molecule Rab5b, F. and the intestinal exosome marker A33. G. Analysis of IL-6 confirms activation of the stress response and specificity of ExoQuick for exosome associated proteins. These data suggest that stress does not impact the rate of exosome release into the plasma. Proteomic results are expressed in means ± SE; 6–8 rats/condition. *indicates significant difference when compared to control rats (p<0.05). #indicates significant difference when compared to exosome depleted fraction (p<0.05). Two-way ANOVA with Fisher PLSD post hoc test was used.

Multiple analyses confirmed exosome enrichment with ExoQuick precipitation. TEM analysis of plasma from stressed rats revealed the presence of exosome-sized particles (∼80 nm) in exosome enriched fractions. The TEM image in Figure 1A shows exosome-sized particles, but the highly stained background and presence of larger, aggregated particles are likely due to fibrins and fibrinogens present in the sample. Figure 1B reveals exosome-sized particles without aggregation, suggesting that treating plasma with Thromboplastin-D prior to ExoQuick fractionation removes aggregating factors [36] for optimal visualization. NTA demonstrated successful isolation of particles 50 to 100 nm under identical scatter conditions for exosome enriched fractions (Figure 1C) from stressed and non-stressed rats (control). These visual observations indicate that ExoQuick treatment of the plasma successfully enriches exosome-sized particles.

ELISA analyses reveal that the exosome associated proteins CD63, Rab5b, and A33 were significantly enriched in exosome enriched plasma fractions from stressed and control rats, compared to their corresponding exosome depleted fractions (Figure 1D–F). IL-6 concentrations, a stress responsive cytokine not known to associate with exosomes, was only significantly elevated in the exosome depleted plasma fraction from stressed rats (Figure 1G), confirming activation of the stress response and successful isolation of proteins only known to associate with exosomes. These data suggest that stressor exposure does not impact the rate of exosome release.

### Stressor exposure increases Hsp72 on the surface of plasma exosomes

**Figure 2:**
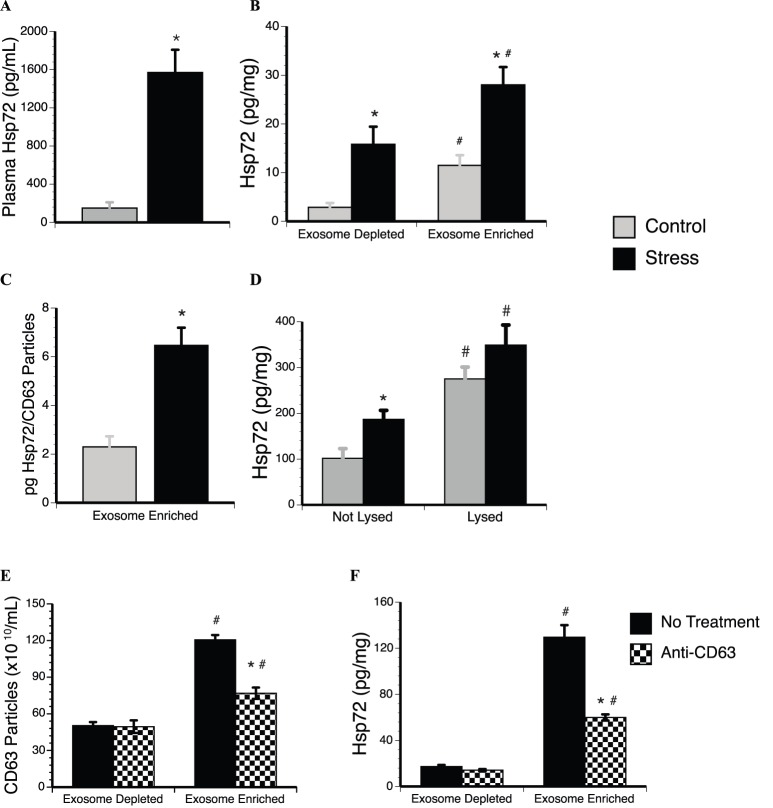
Exposure to an acute stressor modifies the proteomic profile of circulating plasma exosomes. A. Male Fisher 344 rats exposed to inescapable tail shocks (Stress) had elevated plasma concentrations of Hsp72 compared to non-stressed rats (Control). Following exosome enrichment with ExoQuick precipitation, B. concentrations of Hsp72 were the highest in the exosome enriched fractions from stressed rats. C. Correction with CD63 particles confirmed that exposure to stress increases Hsp72 concentrations in the exosome enriched fraction compared to control conditions. D. Lysing the exosome enriched fraction increased Hsp72 concentrations for both the stress and control groups, but the impact of stressor exposure was restricted to the not lysed group. E. Following pre-treatment with Thromboplastin-D, plasma was divided and either treated with magnetic Dynabeads coated in CD63 antibodies (Anti-CD63) to immunoprecipitate CD63 particles from the plasma or left undisturbed (No Treatment) prior to ExoQuick precipitation. Anti-CD63 treatment attenuates CD63 concentrations in the plasma exosome enriched fractions from stressed rats compared no treatment conditions. F. Anti-CD63 treatment also attenuates Hsp72 in the plasma exosome enriched fractions from stressed rats compared to no treatment conditions. Results are expressed in means ± SE; 6–8 rats/condition. *indicates significant difference when compared to control rats (p<0.05). #indicates significant difference when compared to exosome depleted fraction (p<0.05). Two-way ANOVA with Fisher PLSD post hoc test was used.

To determine if stressor exposure modifies the proteomic character of plasma exosomes, we examined the association between stress-inducible Hsp72 and exosomes. Tail shock exposure activated the stress response as indicated by reduction in spleen weights, as well as elevations in plasma corticosterone and blood glucose (Figure S1 in File S1). Hsp72 is highly stress-responsive and, consistent with prior observations, stressed rats had marked accumulation of Hsp72 in the plasma compared to control rats (Figure 2A) [14]. Quantification of Hsp72 following exosome isolation revealed that exposure to stress significantly elevated Hsp72 in both the exosome depleted and exosome enriched plasma fractions compared to corresponding fractions in the control group (Figure 2B), but Hsp72 was significantly elevated in the exosome enriched control conditions compared to the exosome depleted control condition. Statistical analyses indicated a significant interaction between stress and exosome enrichment on Hsp72 concentrations. These data indicate that tail shock stress elevates Hsp72 in both the exosome depleted and exosome enriched fractions, but the highest concentration of Hsp72 is present in the exosome enriched fractions. Further analysis of the exosome enriched fractions revealed that stressed rats also have significantly greater accumulations of Hsp72 compared to control rats when corrected by CD63 concentrations instead of total protein (Figure 2C).

As a first attempt to determine whether the stress-induced elevations of Hsp72 occurs within the lumen of the exosomes or if is restricted to their surface, we also measured Hsp72 after lysing exosomes in the exosome enriched plasma fractions. While lysing increased Hsp72 concentrations in the exosome enriched fractions for both the stress and control groups, there was no impact of stress (Figure 2D). These data suggest that tail shock stress impacts membrane associated Hsp72 on plasma exosomes, but not necessarily lumen-associated Hsp72.

Immunoprecipitation with Dynabeads coated in anti-CD63 antibodies reduced CD63 concentrations in the exosome enriched fraction compared to untreated plasma (Figure 2E). CD63 immunoprecipitation had no impact on CD63 concentration in the exosome depleted fraction. CD63 depletion significantly decreased Hsp72 concentrations in the exosome enriched fraction from the plasma of stressed rats (Figure 2F), indicating that a significant portion of stress-induced plasma Hsp72 is complexed with CD63 positive exosomes.

### Stress reduces exosomal miR-142-5p and miR-203

**Figure 3:**
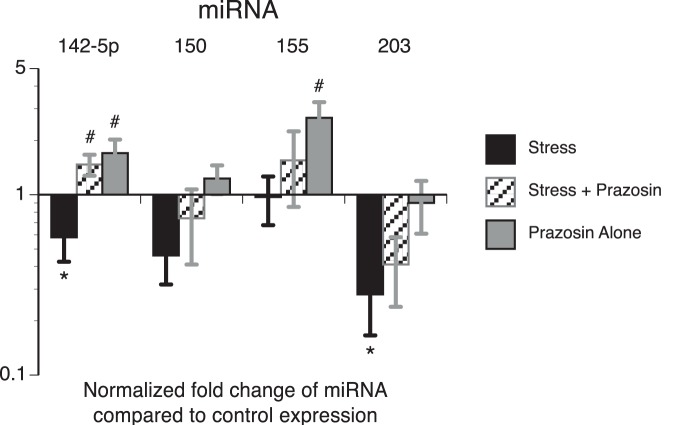
Impact of acute stressor and adrenergic regulation of plasma exosomes miRNA. Exposure to tail shock stress (Stress) significantly reduced plasma exosome miR-142-52 and -203. Intraperitoneal administration of the α_1_-ADR antagonist prazosin (2.0 mg/kg) 30 min prior to Stress attenuated the stress-induced down-regulation of miR-142-5p, but not miR-203. *indicates significant fold change when compared to plasma exosomes from control rats in the absence of prazosin (p<0.05). #indicates significant effect of prazosin (p<0.05). Two-way ANOVA with Fisher PLSD post hoc test was used.

Plasma exosomes isolated from the α_1_-ADR study were analyzed for miRNA related to stress and inflammation (miR-142-5p, -150, -155, and -203) and variations were observed. Exosomal miR-142-5p and -203 were significantly reduced in stressed rats compared to exosomes isolated from control rats (Figure 3). To our knowledge, this is the first evidence that *in* *vivo* exposure to an acute stressor, in the absence of pathogens or disease, modifies miRNA content in circulating plasma exosomes.

### Pathway enrichment analyses of rno-miR-142-5p and rno-miR-203

Based on miRDB.com query, 374 genes are predicted targets of rno-miR-142-5p and 386 genes are predicted targets of rno-miR-203. These targets were uploaded into the WebGestaldt Bioinformatics system and subjected to KEGG and Wiki pathway analyses. Of these 760 total predicted gene targets, 736 were unambiguously mapped and included in the analyses. Table 1 exhibits the pathways identified by KEGG and Wiki modulated by the rno-miR-142-5p and rno-miR-203. The number of genes in each pathway and a *p*-value associated with each functional pathway category is reported. KEGG and Wiki analyses revealed that rno-miR-142-5p and rno-miR-203 are related to several functional categories, including metabolic pathways (25 genes), mRNA processing (12 genes), cancer pathways (10 genes), ubiquitin mediated proteolysis (9 genes), mitogen-activated protein kinase (MAPK) signaling (8 genes), transforming growth factor-β (TGF-β) signaling pathway (6 genes), and TGF-β receptor signaling pathway (6 genes).

**Table 1:** Functionally enriched pathways impacted by miR-142-5p and miR-203.

Bioinformatics Database *Functionally Enriched Pathway* rno-miR-142-5p	Gene Count	Adj. P value
**KEGG**		
*Ubiquitin mediated proteolysis*	9	0.0001
*Pathways in cancer*	10	0.0127
*Spliceosome*	6	0.0227
*VEGF signaling pathway*	4	0.0263
*Renal cell carcinoma*	4	0.0263
*Wnt signaling pathway*	6	0.0263
*Carbohydrate digestion and absorption*	3	0.0263
*Cell cycle*	5	0.0263
*Gastric acid secretion*	4	0.0263
*Phosphatidylinositol signaling system*	4	0.0263
**WIKI**		
*Proteasome degradation*	*6*	*0.0003*
*mRNA processing*	*7*	*0.0018*
*Kit receptor signaling pathway*	*5*	*0.0040*
**rno-miR-203**	**Gene Count**	**Adj. P value**
**KEGG**		
*Metabolic Pathways*	25	0.0018
*TGF-beta signaling pathway*	6	0.0034
*Aldosterone-regulated sodium reabsorption*	4	0.0140
*Fc gamma R-mediated phagocytosis*	5	0.0168
*Insulin signaling pathway*	6	0.0168
*MAPK signaling pathway*	8	0.0280
*RNA degradation*	4	0.0343
*Hypertrophic cardiomyopathy (HCM)*	4	0.0343
*Phosphatidylinositol signaling system*	4	0.0343
*Focal adhesion*	6	0.0343
**WIKI**		
*MAPK signaling pathway*	7	0.0215
*Diurnally regulated genes with circadian orthologs*	3	0.0430
*NFE2L2*	2	0.0430
*P53 pathway*	3	0.0430
*mRNA processing*	5	0.0430
*Adipogenesis*	5	0.0430
*Insulin signaling*	6	0.0430
*Wnt signaling pathway*	5	0.0430
*Small ligand GPCrs*	2	0.0463
*TGF-beta receptor signaling pathway*	6	0.0473

KEGG and Wikipathway (Wiki) analysis revealed functionally enriched pathway categories generated from genes significantly differentially represented in plasma exosomes from rats exposed to inescapable tail shock.

### Prazosin attenuates stress-modified changes in exosomal Hsp72 and miR-142-5p

**Figure 4:**
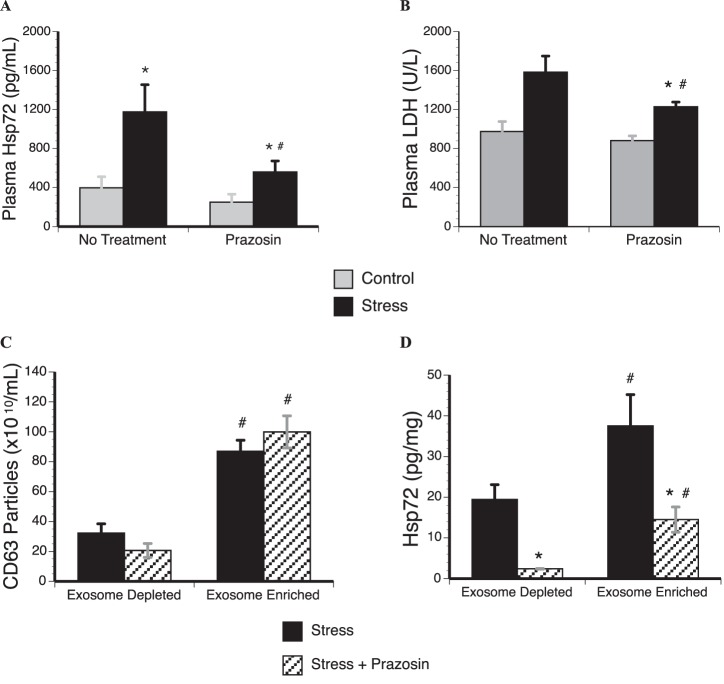
Adrenergic regulation of exosomal Hsp72. Adult male Fisher 344 rats were either injected intraperitoneally with the α_1_-ADR antagonist prazosin (2.0 mg/kg) 30 min prior to exposure to tail shock stress (Stress) or left undisturbed. Plasma analysis reveals that prazosin administration A. significantly attenuates stress-induced elevations of Hsp72 as well as B. lactate dehydrogenase activity, a marker of cell death. Exosomes were fractionated with ExoQuick in the stressed rats, revealing that prazosin administration C. had no effect on CD63 particle concentrations, but D. significantly attenuates Hsp72 concentration in both the exosome depleted and exosome enriched fractions. Results are expressed in means ± SE; 6–8 rats/condition. *indicates significant difference when compared to control rats (p<0.05). #indicates significant difference when compared to no treatment group or exosome depleted fraction (p<0.05). Two-way ANOVA with Fisher PLSD post hoc test was used.

Consistent with previous findings, prazosin administration in stressed rats reduced plasma concentrations of Hsp72 compared to untreated rats exposed to the same stressor (Figure 4A) while boosting stress-induced elevations in blood glucose and attenuating stress-induced reductions in spleen weight (Figure S2 in File S1) [35]. Prazosin did not impact plasma Hsp72, spleen weight, or blood glucose concentrations in the control rats, confirming prazosin selectivity to sympathetic nervous system (SNS) activity [35], [37], [38]. Prazosin administration attenuated stress-induced cell death, as quantified by LDH (Figure 4B). Prazosin had no impact on CD63 (Figure 4C) or total protein (Figure S2 in File S1); however, α_1_-ADR blockade significantly decreased Hsp72 concentrations in both exosome fractions (Figure 4D). Additionally, reductions in miR-142-5p were reversed with prazosin administration (Figure 3).

## Discussion

A variety of disease states can change both the composition and function of exosomes. What remains less clear is if exosomal modifications occur during the acute stress response. In this series of experiments, we reveal that exposure to an intense and acute stressor in the absence of injury or disease alters the proteomic and miRNA composition of plasma exosomes. Additionally, this stress-induced modification of plasma exosomes is partially mediated by SNS activation of α_1_-ADRs.

Circulating Hsp72 is increased following exposure to a variety of acute stressors [14], [35] and the release of the protein may contribute to stress-modulated immunity [16], [39] through activation of macrophages, dendritic cells, and neutrophils, inducing the secretion of pro-inflammatory cytokines [40]. Here we provide evidence that a significant portion of stress-induced Hsp72 is released into the blood via an exosome release pathway. CD63 immunoprecipitation of plasma from stressed rats prior to exosome isolation corresponds to a marked decrease in Hsp72, further supporting its association with circulating exosomes. Analysis of Hsp72 in the exosome enriched fractions following lysing revealed that while Hsp72 is present within the exosome lumen, the stress-induced changes in exosomal Hsp72 are restricted to the surface. Consistent with the exosome literature, our data indicate that exosome-associated Hsp72 is released, albeit at low levels, during basal states [19], [20], [23]. Stressor exposure also elevates Hsp72 in the exosome depleted plasma fractions, suggesting that a portion of circulating Hsp72 is soluble, perhaps released into the blood after necrotic cell death [14]. Additionally, there is evidence that enzymes in the blood may cleave exosome associated proteins [41], potentially releasing soluble Hsp72 from stress-modified plasma exosomes. Together, these findings demonstrate that circulating exosomes in stressed rats have higher concentrations of surface Hsp72 compared to non-stressed control rats, and the presence of soluble Hsp72 could be the result of either stress-induced cell death or enzymatic cleavage of the exosomal proteins.

The data presented here also demonstrate that exposure to an acute stressor down-regulates miR-142-5p and -203 in circulating plasma exosomes, likely affecting macrophage and dendritic cell-mediated immunity. Recent findings indicate that down-regulation of miR-142 promotes early normal macrophage differentiation [30]. Down-regulation of miR-142-5p is also known to enable cytokine-mediated survival signaling in response to hemodynamic stress through the IL-6 receptor, gp130 [27], further supporting its immunological contribution during the stress response. Additionally, miR-203 is known to directly target suppressor of cytokine signaling 3 (SOCS3) and block its role as an inhibitor of cytokine signal transduction [42]. Activation of SOCS3 terminates the innate immune response; therefore, stress-induced reductions in exosomal miR-203 could prevent activation of SOCS3 and its downstream suppression of pro-inflammatory cytokines. Down-regulation of miR-203 is also capable of inducing TNF-α and IL-6 synthesis by enabling myeloid differentiating factor 88 (MyD88) activation [43].

Pathway analyses of the 760 predicted mRNA targets of miR-142-5p and -203 revealed functionally enriched pathways that overlap with demonstrated targets of the stress response. For example, exposure to the same stressor tested here is known to modulate genes involved in the TGF-β pathway [44], which is a prime target of miR-203. In addition, miR-203 is known to repress the anti-inflammatory properties of TGF-β [45]. Therefore, stress-induced reductions in miR-203 likely promote elevations in TGF-β activity, which may be crucial for constraining stress-induced immunomodulation. KEGG analysis also revealed that miR-203 targets metabolic pathways, which is in line with cortisol mediation of metabolic pathways during the stress response [46]. Additional KEGG analysis of predicted gene targets for miR-142-5p revealed involvement with ubiquitin mediated proteolysis. Reductions in miR-142 are associated with elevations in ubiquitin proteasome activity [47], which is a cellular strategy for degrading proteins denatured by stress and repairing DNA damage [48], Collectively, these observations suggest that stress-induced down-regulation of miR-142-5p and -203 and up-regulation of Hsp72 may be crucial components of exosome-based stress-induced immunomodulation and cytoprotection, which is consistent with previous findings demonstrating the immunomodulating and cellular consequences of stress [39], [49]. Future studies should examine the impact of stress-modified exosomes on these gene targets in cell lines, which would provide valuable insight into the role of exosomal miRNA during the stress response.

This study also reveals that exosomal modification in Hsp72 and miR-142-5p from stressed rats rely on sympathetic nervous system release of norepinephrine (NE) and its subsequent activation of one of its target receptors, the α_1_-ADR. Consistent with previous research, blockade of the α_1_-ADR with prazosin prior to inescapable tail shock stress reduces stress-induced elevations of Hsp72 in plasma [35], [50], and attenuates the stress-induced down-regulation of miR-142-5p. Since down-regulation of miR-142-5p is known to enable cytokine-mediated survival [27], prazosin administration should decrease associated inflammatory cytokine activity. Indeed, previous research demonstrates that pre-treatment with prazosin prior to tail shock stress attenuates stress-induced elevations in monocyte chemotactic protein-1 (MCP-1) and IL-1β [51]. In line with these findings, administration of phenylephrine, an α_1_-ADR agonist, in the absence of stress induces an elevation of plasma Hsp72 similar to levels seen in rats exposed to inescapable tail shock [35]; however, additional studies are needed to determine if stimulation of the α_1_-ADR in the absence of an acute stressor modifies exosomal Hsp72 and miRNAs. Interestingly, prazosin administration also decreased Hsp72 in the exosome depleted fraction of stressed rats. LDH assessment of the plasma reveals that prazosin administration attenuates cell death, which likely decreases the necrotic release of soluble Hsp72 into the circulation. While both NE and epinephrine (E) bind to α_1_-ADRs, NE has a higher affinity for these receptors and depletion of E through adrenalectomy has no effect on stress-induced Hsp72 in the circulation [35].

Based on these findings, we hypothesize that exposure to an intense, acute stressor modifies plasma exosome cargo, specifically Hsp72 and miR-142-5p, by activating the SNS and inducing the release of NE from sympathetic nerve terminals. Stimulation of the α_1_-ADRs by NE activates phospholipase C and elevates cytosolic Ca^2+^. Fusion of the multivesicular body (MVB), the endocytic source of exosomes, to the plasma membrane is Ca^2+^ dependent; therefore, the surge in Ca^2+^ may facilitate exosome release. Alternatively, NE stimulation of ADRs increases ubiquitination through Ca^2+^ flux [52], which is required for targeting cellular proteins to endosomes prior to fusing with the MVB. Interestingly, the presence of multiple exosome markers, such as the tetraspanin CD63, the membrane transport protein Rab5b, and the intestinal epithelial exosome marker A33, were unchanged following exposure to inescapable tail shock. Additionally, CD63 concentrations are unaffected by prazosin, therefore it is unlikely that activation of the α_1_-ADR impacts exosome release, but rather their composition through ubiquitination. Thus, exposure to an acute stressor potentially modifies exosome-associated Hsp72 and miRNA in the plasma by increasing their rate of loading onto intracellular endosomes rather than impacting the rate of secretion. Conversely, α_1_-ADR activation may be critical for Hsp72 synthesis or miRNA transcription. A recent study demonstrated that blockade of the α_1_-ADR in stressed rats attenuated stress-induced increases of intracellular Hsp72 in the spleen, liver, and subcutaneous adipose [51], however, it is unknown if these are the tissue source for plasma exosome Hsp72. Future studies should elucidate the tissue origin of these stress modified exosomes and examine if α_1_-ADR blockade in stressed animals affects their cytosolic Hsp72 and miR-142-5p, thus indicating whether α_1_-ADR activation is critical for their transcription and synthesis or their exosomal loading.

The release of Hsp72 and miRNAs through an exosomal pathway has several advantages. Most notably, exosomes provide a protective lipid bi-layer that can facilitate long distance communication between cells. From a stress physiology perspective, this form of cellular communication may be evolutionarily advantageous. For example, if an organism is subjected to a harmful stressor, such as a predator, the organism’s cells could secrete stress-modified exosomes into the circulation prior to experiencing injury. When injury occurs, stress-modified exosomes are already in the circulation and available to facilitate the host immune response. Cytokine induction by resident leukocytes at the site of injury triggers the activation and expression of adhesion molecules on the adjacent vascular endothelium. Since intercellular adhesion molecules (ICAMs) are present on exosomes [5], plasma exosomes could bind ICAM receptors on vascular endothelial cells and consequently leave the circulation and migrate to the injured tissue. At the site of infection, stress-modified exosomes could boost innate immunity through Hsp72-mediated TLR4 activation of macrophages and neutrophils, or transfer their content through clathrin-mediated endocytosis with a recipient cell, subsequently stimulating a pro-inflammatory cytokine response and enhancing the organism’s chance of survival.

There are a variety of clinical applications for stress-modified exosomes that could potentially modulate immunity. For example, exposing cells to a non-lethal stressor, such as heat, to elevate Hsp72 and down-regulate miR-142-5p and -203 in exosomes could enhance the immunogenicity of exosome-based vaccines, specifically cancer vaccines [8], [53]. Alternatively, since exosomes are capable of delivering their content to recipient cells [25], they could transfer their content to target cells, where Hsp72 could translocate to the target cell’s cytosol and perform its cytoprotective and anti-apoptotic functions and miRNAs could modulate mRNA translation.

In summary, our data indicate that *in* *vivo* exposure to an acute stressor modifies the proteomic and miRNA character of exosomes released into the plasma, likely impacting innate immune function through TLR association, monocyte differentiation, and cytokine secretion. Furthermore, our results suggest that SNS activation of α_1_-ADRs is a critical component of some of these exosomal modifications. Given the known immunomodulatory and protective functions of Hsp72, miRNA, and exosomes, we speculate that modulation of plasma exosomes is a critical component of the stress response. Future studies should further identify the immunomodulatory factors and cellular sources of stress-modified exosomes in the plasma, which will challenge current paradigms concerning the mechanisms of stress-evoked modulation of immunity and advance knowledge concerning their use in immunotherapy.

## Supporting Information

File S1 Figure S1, Inescapable tail shock activates the stress response. Figure S2, Alpha-1 adrenergic regulation of blood glucose, spleen weight, and total protein.
(DOCX)
