# Supplementary material for: Acute Stressor Exposure Modifies Plasma Exosome-Associated Heat Shock Protein 72 (Hsp72) and microRNA (miR-142-5p and miR-203)
Source: PLoS One. 2014 Sep 26;9(9):e108748. doi: 10.1371/journal.pone.0108748 (PMC4178201; doi:10.1371/journal.pone.0108748)
Supplement: File S1 — Figure S1, Inescapable tail shock activates the stress response. Figure S2, Alpha-1 adrenergic regulation of blood glucose, spleen weight, and total protein. (DOCX) [file pone.0108748.s001.docx]

**SUPPLEMENTAL DATA**

Beninson et al.

**Figure S1. Inescapable tail shock activates the stress response.**

**A B**

**C**

**Figure S1. Inescapable tail shock activates the stress response.** Male Fisher 344 rats exposed to inescapable tail shock (Stress) activates the stress response as indicated by **A.** reduction in spleen weight, **B.** elevated plasma corticosterone, and **C.** elevated blood glucose compared to non-stressed controls. Results are expressed in means ± SE; 6-8 rats/condition. *Indicates significant difference when compared to control rats (p < 0.05). Two-way ANOVA was used.

**Figure S2. Alpha-1 adrenergic regulation of blood glucose, spleen weight, and total protein.**

**A B**

**C**

**Figure S2. Alpha-1 adrenergic regulation of blood glucose, spleen weight, and total protein.** Adult male Fisher 344 rats were either injected intraperitoneally with the α_1_-adrenergic receptor antagonist prazosin (2.0 mg/kg) 30 minutes prior to exposure to tail shock stress (Stress) or left undisturbed. Successful prazosin administration was confirmed by **A.** elevations blood glucose and **B.** elevations in spleen/body weight percentage in stressed rats. **C.** Prazosin administration in the stressed rats had no effect on exosome enriched or exosome depleted concentrations of BCA. Results are expressed in means ± SE; 6-8 rats/condition. * indicates significant difference when compared to control rats (p < 0.05). # indicates significant difference when compared to no treatment group or exosome depleted fraction (p < 0.05). Two-way ANOVA with Fisher PLSD post hoc test was used.
